# Supplementary material for: Glucose-6-phosphate dehydrogenase maintains redox homeostasis and biosynthesis in LKB1-deficient KRAS-driven lung cancer
Source: Nat Commun. 2024 Jul 12;15:5857. doi: 10.1038/s41467-024-50157-8 (PMC11245543; doi:10.1038/s41467-024-50157-8)
Supplement: Supplementary file 1 — Supplementary information [file 41467_2024_50157_MOESM1_ESM.pdf]

Supplementary information for

Glucose-6-Phosphate Dehydrogenase Maintains Redox Homeostasis and Biosynthesis in *LKB1*-Deficient *KRAS*-Driven Lung Cancer
Taijin Lan[1], Sara Arastu[1], Jarrick Lam[1], Hyungsin Kim[1], Wenping Wang[1], Samuel Wang[1], Vrushank Bhatt[1], Eduardo Cararo Lopes[1,2], Zhixian Hu[1], Michael Sun[1], Xuefei Luo[1], Jonathan M. Ghergurovich[3], Xiaoyang Su[1,4], Joshua D. Rabinowitz[1,5,6,7], Eileen White[1,2,6], Jessie Yanxiang Guo[1,4,8,*]

[1]Rutgers Cancer Institute, New Brunswick, New Jersey 08901, USA
[2]Department of Molecular Biology and Biochemistry, Rutgers University, Piscataway, New Jersey 08854, USA
[3]Department of Molecular Biology, Princeton University, Princeton, New Jersey 08544, USA
[4]Department of Medicine, Rutgers Robert Wood Johnson Medical School, New Brunswick, New Jersey 08901, USA
[5]Department of Chemistry, Princeton University, Princeton, New Jersey 08544, USA
[6]Ludwig Princeton Branch, Ludwig Institute for Cancer Research, Princeton University, Princeton, New Jersey 08540, USA.
[7]Lewis-Sigler Institute of Integrative Genomics, Princeton University, Princeton, NJ, USA.
[8]Department of Chemical Biology, Rutgers Ernest Mario School of Pharmacy, Piscataway, New Jersey 08854, USA
* Corresponding Author

Corresponding Author:
Jessie Yanxiang Guo, Ph.D.
Rutgers Cancer Institute
RBHS-Robert Wood Johnson Medical School
Rutgers, The State University of New Jersey
195 Little Albany Street
New Brunswick, NJ 08901
yanxiang@cinj.rutgers.edu
Voice: 732-235-9657

Supplementary Fig. 1

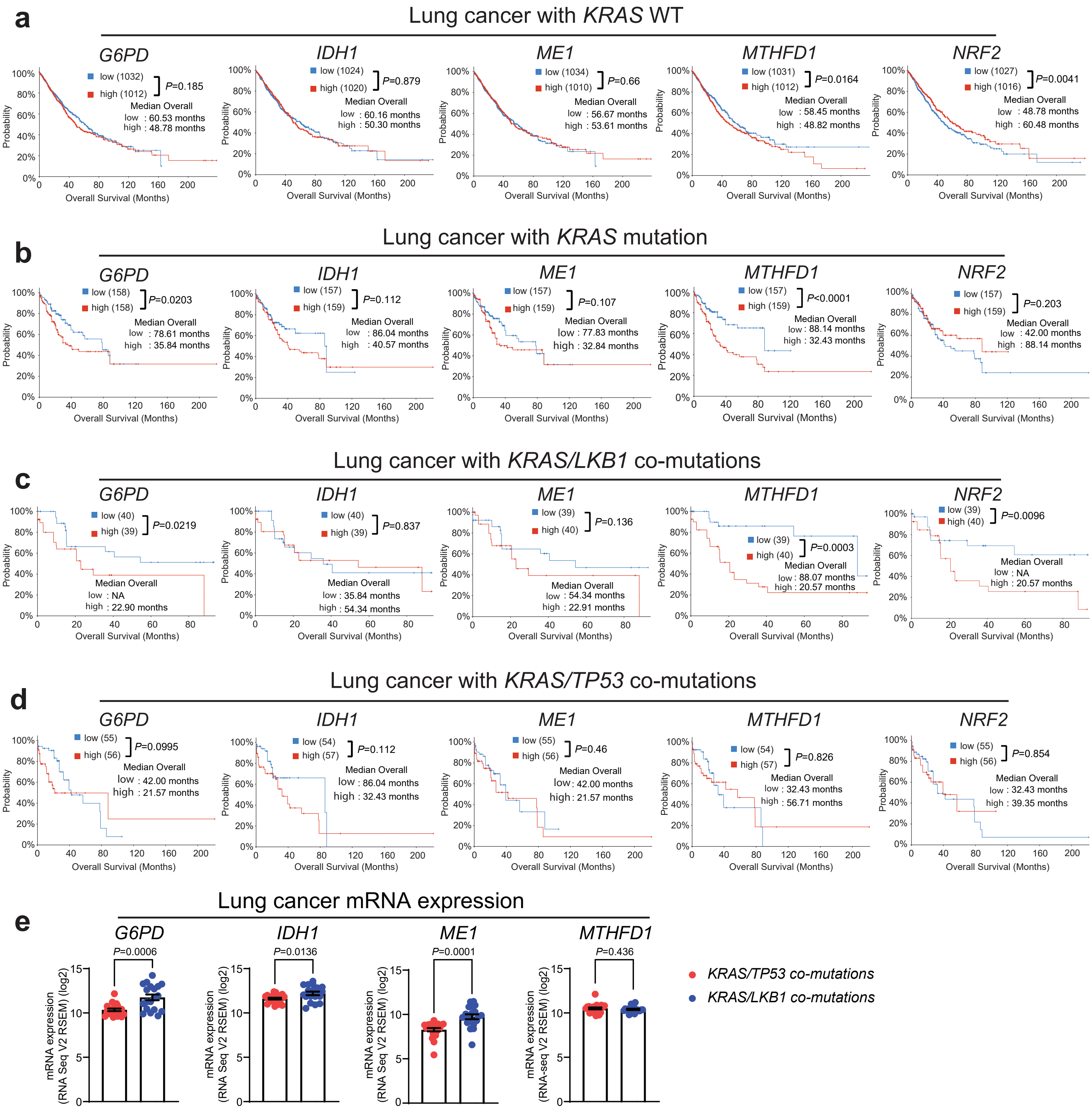

Supplementary Fig. 1: High *G6PD* expression correlates with poor survival of lung cancer patients with *KRAS/LKB1* co-mutations.

a. Overall survival comparison between low and high mRNA expression group of *G6PD*, *IDH1*, *ME1*, *MTHFD1*, and *NRF2* in lung cancer patients with *KRAS* wild type (WT), using data obtained from cBioPortal datasets on December 09, 2023. n = 2044 patients for G6PD, IDH1, ME1, MTHFD1, n = 2043 patients for NRF2.
b. Overall survival comparison between low and high mRNA expression group of *G6PD*, *IDH1*, *ME1*, *MTHFD1*, and *NRF2* in lung cancer patients with *KRAS* mutation, using data obtained from cBioPortal datasets on December 09, 2023. n = 316 patients for each gene.
c. Overall survival comparison between low and high mRNA expression group of *G6PD*, *IDH1*, *ME1*, *MTHFD1*, and *NRF2* in lung cancer patients with *KRAS/LKB1* co-mutations, using data obtained from cBioPortal datasets on December 09, 2023. n = 79 patients for each gene.
d. Overall survival comparison between low and high mRNA expression group of *G6PD*, *IDH1*, *ME1*, *MTHFD1*, and *NRF2* in lung cancer patients with KRAS/TP53 co-mutations, using data obtained from cBioPortal datasets on December 09, 2023. n = 111 patients for each gene.
e. The mRNA expression levels of *G6PD*, *ME1*, *IDH1*, and *MTHFD1* in lung cancer patients with *KRAS/TP53* co-mutations (n = 22 patients) and KRAS/LKB1 co-mutations (n = 19 patients), using data obtained from cBioPortal datasets on December 09, 2023.

Data are presented as mean ± SEM, significance was performed by cBioPortal (version 6.0.1). Source data are provided as a Source Data file.

Supplementary Fig. 2

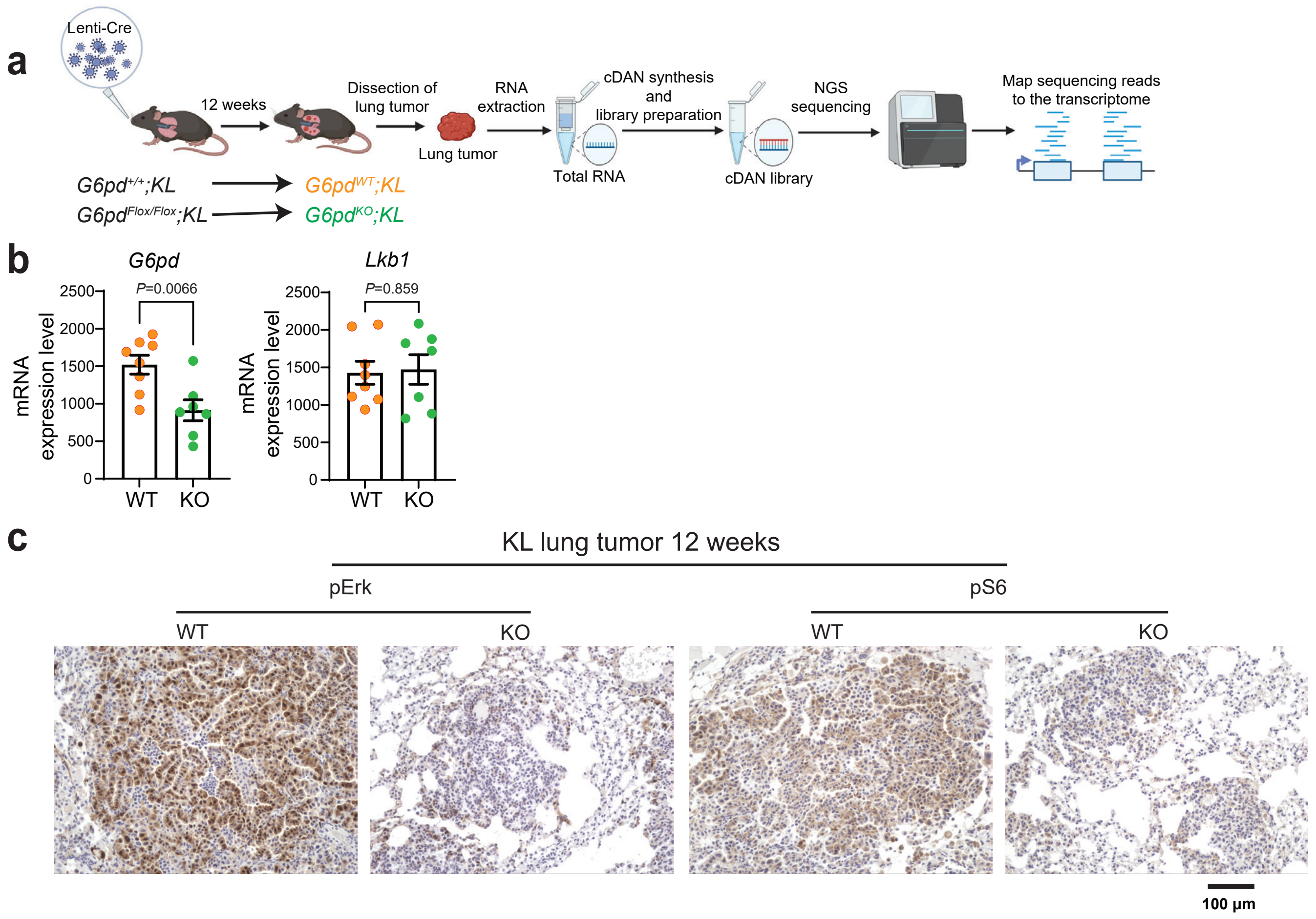

Supplementary Fig. 2: *G6pd* and *Lkb1* mRNA expression from bulk tumor RNA-seq and IHC staining of pErk and pS6 of KL lung tumors.

a. Scheme illustrating the bulk tumor RNA-seq of $G6pd^{WT}$*;KL* and $G6pd^{KO}$*;KL* lung tumors at 12 weeks post-tumor induction (Created with BioRender.com released under a Creative Commons Attribution-NonCommercial-NoDerivs 4.0 International license).

b. *G6pd* and *Lkb1* mRNA expression of $G6pd^{KO}$*;KL* (n = 7 mice) and $G6pd^{WT}$*;KL* (n = 8 mice) lung tumors.

c. Lower magnification of IHC staining of pERK (n = 24 images for $G6pd^{WT}$*;KL*, n = 12 images for $G6pd^{KO}$*;KL*) and pS6 (n = 18 images for $G6pd^{WT}$*;KL*, n = 15 images for $G6pd^{KO}$*;KL*) of KL lung tumors at 12 weeks post-tumor induction to show reduced growth signaling of KL lung tumors by G6PD depletion.

Data are presented as mean ± SEM, significance was calculated by two-tailed unpaired *t*-test (b). Source data are provided as a Source Data file.

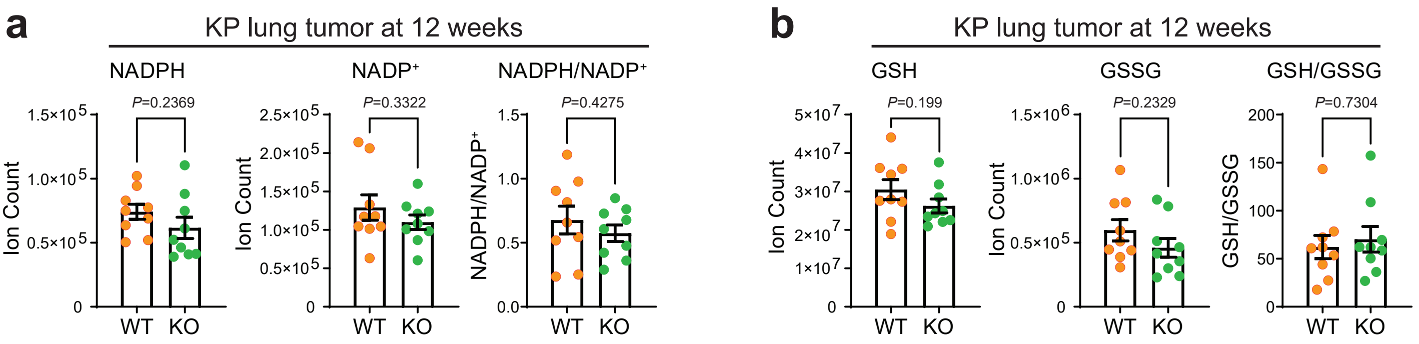
a
KP lung tumor at 12 weeks
NADPH
NADP⁺
NADPH/NADP⁺
P=0.2369
P=0.3322
P=0.4275
Ion Count
NADPH/NADP⁺
WT KO
b
KP lung tumor at 12 weeks
GSH
GSSG
GSH/GSSG
P=0.199
P=0.2329
P=0.7304
Ion Count
GSH/GSSG
WT KO

Supplementary Fig. 3: G6PD loss has no effect on KP lung tumor redox homeostasis.

a. Pool size of NADPH and NADP+, and NADPH/NADP+ ratio in *G6pd*^{WT}*;KP* (n = 9 mice) and *G6pd*^{KO}*;KP* (n = 9 mice) lung tumors at 12 weeks post-tumor induction.

b. Pool size of glutathione (GSH) and glutathione disulfide (GSSG), and GSH/GSSG ratio in *G6pd*^{WT}*;KP* (n = 9 mice) and *G6pd*^{KO}*;KP* (n = 9 mice) lung tumors at 12 weeks post-tumor induction.

Data are presented as mean ± SEM, significance was calculated by two-tailed unpaired *t*-test (a, GSH and GSSG in panel b), or Mann Whitney test (GSH/GSSG in panel b). Source data are provided as a Source Data file.

Supplementary Fig. 4

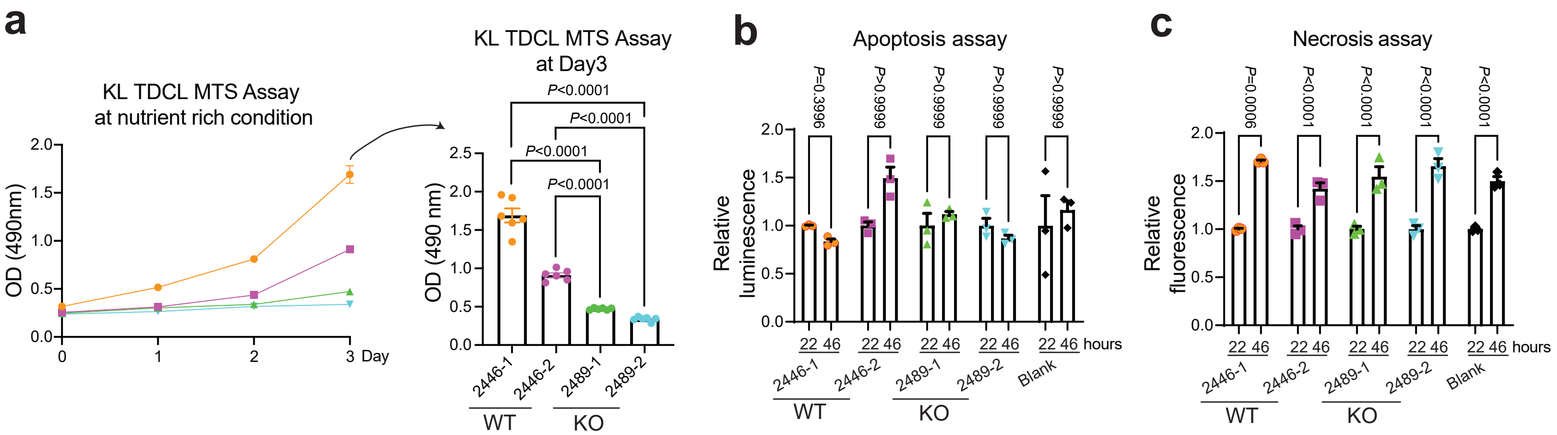

Supplementary Fig. 4: Reduced *G6pd*^{KO}*;KL* TDCLs proliferation is independent of apoptosis and necrosis.

a. MTS assay of *G6pd*^{WT}*;KL* and *G6pd*^{KO}*;KL* TDCLs in nutrient rich conditions. n = 6 replicates for each clone at indicated timepoint, except 2489-2 with n = 5 replicates at day 0 and n = 6 replicates at day 1, 2, and 3.

b & c. Apoptosis (b) and necrosis (c) assay of *G6pd*^{WT}*;KL* and *G6pd*^{KO}*;KL* TDCLs in nutrient rich conditions. n = 3 replicates for each clone at indicated timepoint.

Data are presented as mean ± SEM, significance was calculated by one-way ANOVA followed by Bonferroni's multiple comparisons test (a, b, c). Source data are provided as a Source Data file.

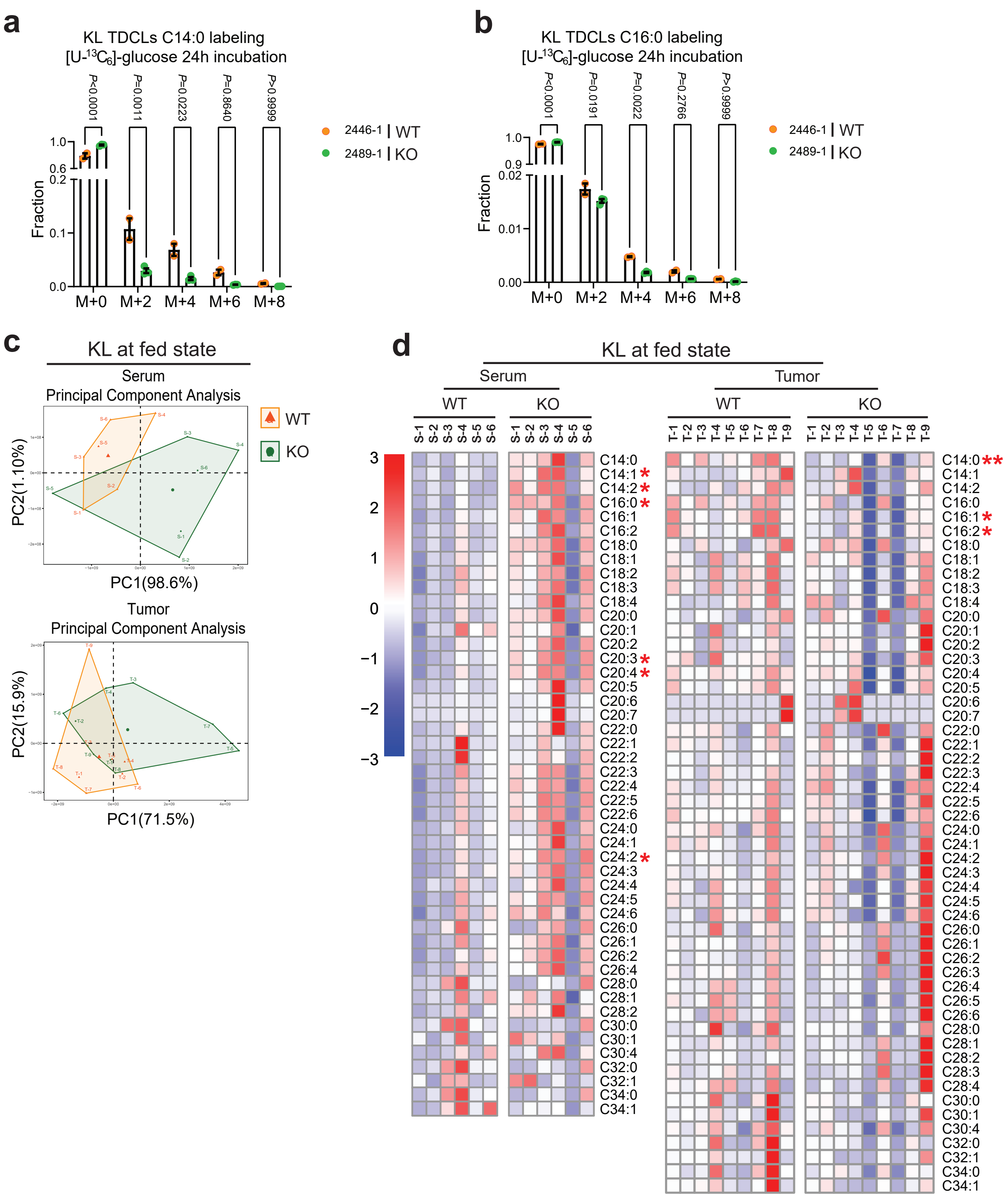
a
KL TDCLs C14:0 labeling
[U-13C6]-glucose 24h incubation
P<0.0001
P=0.0011
P=0.0223
P=0.8640
P>0.9999
Fraction
M+0
M+2
M+4
M+6
M+8
2446-1 | WT
2489-1 | KO
b
KL TDCLs C16:0 labeling
[U-13C6]-glucose 24h incubation
P<0.0001
P=0.0191
P=0.0022
P=0.2766
P>0.9999
Fraction
c
KL at fed state
Serum
Principal Component Analysis
PC1(98.6%)
PC2(1.10%)
WT
KO
Tumor
Principal Component Analysis
PC1(71.5%)
PC2(15.9%)
d
KL at fed state
Serum
Tumor
WT
KO
C14:0
C14:1
C14:2
C16:0
C16:1
C16:2
C18:0
C18:1
C18:2
C18:3
C18:4
C20:0
C20:1
C20:2
C20:3
C20:4
C20:5
C20:6
C20:7
C22:0
C22:1
C22:2
C22:3
C22:4
C22:5
C22:6
C24:0
C24:1
C24:2
C24:3
C24:4
C24:5
C24:6
C26:0
C26:1
C26:2
C26:3
C26:4
C26:5
C26:6
C28:0
C28:1
C28:2
C28:3
C28:4
C30:0
C30:1
C30:4
C32:0
C32:1
C34:0
C34:1

Supplementary Fig. 5: G6PD deficiency affects KL lung tumor lipid metabolism.

a & b. C14:0 (a) and C16:0 (b) ^{2}H labeling fraction of *G6pd*^{WT}*;KL* and *G6pd*^{KO}*;KL* TDCLs after 24 hours [U-$^{13}C_6$]-glucose labeling. n = 2 replicates for *G6pd*^{WT}*;KL* TDCL, n = 3 replicates for *G6pd*^{KO}*;KL* TDCL.

c & d. Principal Component Analysis (PCA) (c) and Heatmap (d) of saponified fatty acid pool size of *G6pd*^{WT}*;KL* and *G6pd*^{KO}*;KL* lung tumors and serum from KL tumor bearing mice in fed state (food was kept in cages, and mice were euthanized with samples collected at 8:00AM) at 12 weeks post-tumor induction. n = 6 mice for tumor and serum samples.

Data are presented as mean ± SEM, significance was calculated by two-tailed unpaired *t*-test (c, d) or two-way ANOVA followed by Bonferroni's multiple comparisons test (a, b). *$P < 0.05$; **$P < 0.01$. Source data are provided as a Source Data file.

Supplementary Fig. 6

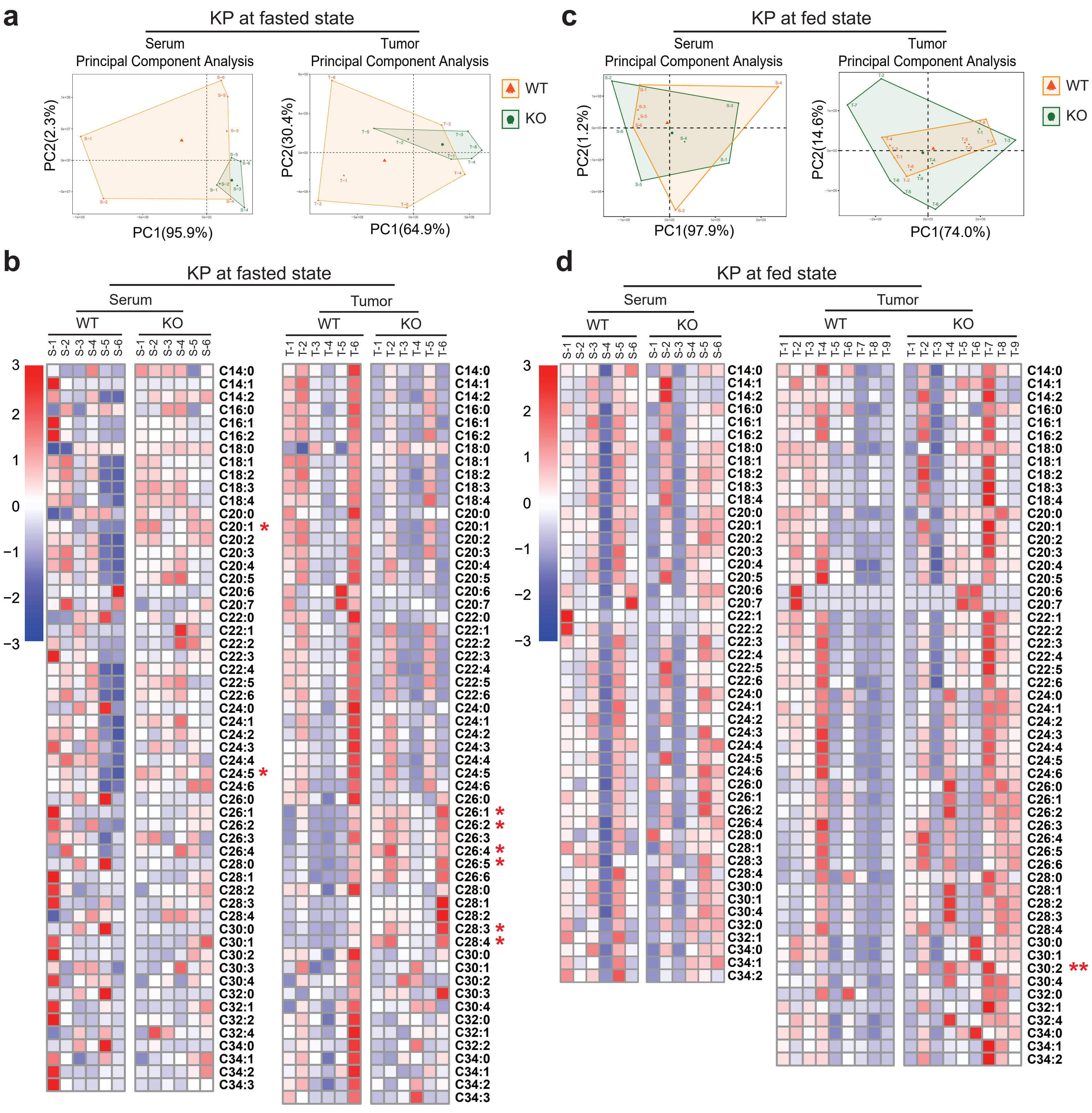

Supplementary Fig. 6: G6PD deficiency does not impact KP lung tumor lipid metabolism.

a & b. Principal Component Analysis (PCA) (a) and Heatmap (b) of saponified fatty acid pool size of *G6pd*^{WT}*;KP* and *G6pd*^{KO}*;KP* lung tumors and serum from KP lung tumor bearing mice in fasted state (food was removed from the mice at approximately 9:00AM, and mice were euthanized with samples collected at 3:00PM) at 12 weeks post-tumor induction. n = 6 mice for tumor and serum samples.

c & d. Principal Component Analysis (PCA) (c) and Heatmap (d) of saponified fatty acid pool size of *G6pd*^{WT}*;KP* and *G6pd*^{KO}*;KP* lung tumors and serum from KP lung tumor bearing mice in fed state (food was kept in cages, and mice were euthanized with samples collected at 8:00AM) at 12 weeks post-tumor induction. n = 6 mice for tumor and serum samples.

Data are presented as mean ± SEM, significance was calculated by two-tailed unpaired *t*-test (a, b, c, d). *$P< 0.05$; **$P< 0.01$. Source data are provided as a Source Data file.

Supplementary Fig.7

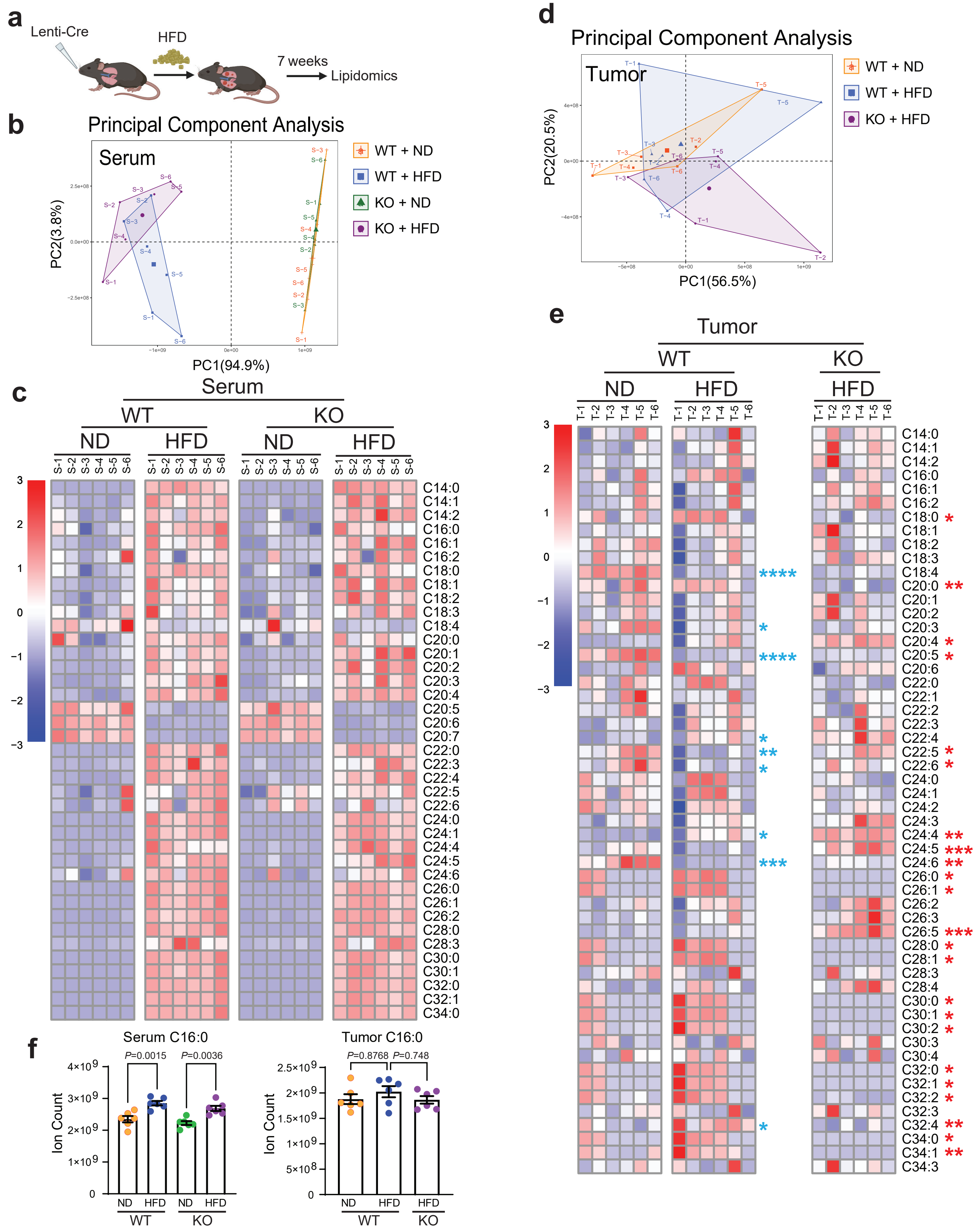

Supplementary Fig. 7: HFD partially rescues the alterations in fatty acyl group composition resulting from G6PD loss.

a. Scheme to examine the effect of HFD on fatty acyl group composition of KL lung tumors (Created with BioRender.com released under a Creative Commons Attribution-NonCommercial-NoDerivs 4.0 International license).

b & c. PCA (a) and heatmap (b) of saponified fatty acid pool size of serum from *G6pd*^{WT}*;KL* or *G6pd*^{KO}*;KL* lung tumor bearing mice fed with normal diet (ND, for 7 weeks) or high fat diet (HFD, for 7 weeks) in fasted state (food was removed from the mice at approximately 9:00AM, and mice were euthanized with samples collected at 3:00PM) at 7 weeks post-tumor induction. n = 6 mice for each group.

d & e. PCA (c) and heatmap (d) of saponified fatty acid pool size of *G6pd*^{WT}*;KL* or *G6pd*^{KO}*;KL* lung tumors from tumor bearing mice fed with normal diet (ND, for 7 weeks) or high fat diet (HFD, for 7 weeks) in fasted state (food was removed from the mice at approximately 9:00AM, and mice were euthanized with samples collected at 3:00PM) at 7 weeks post-tumor induction. n = 6 mice for each group. Blue asterisks denote significance between ND and HFD in *G6pd*^{WT}*;KL* tumors, while red asterisks denote significance between *G6pd*^{WT}*;KL* and *G6pd*^{KO}*;KL* tumors in HFD.

f. Pool size of C16:0 of *G6pd*^{WT}*;KL* and *G6pd*^{KO}*;KL* tumors and serum from (b) and (d).

Data are presented as mean ± SEM, significance was calculated by two-tailed unpaired *t*-test (d) one-way ANOVA followed by Bonferroni's multiple comparisons test (e). $^{*}P< 0.05$; $^{**}P< 0.01$; $^{***}P< 0.001$; $^{****}P< 0.0001$. Source data are provided as a Source Data file.

Supplementary Fig. 8

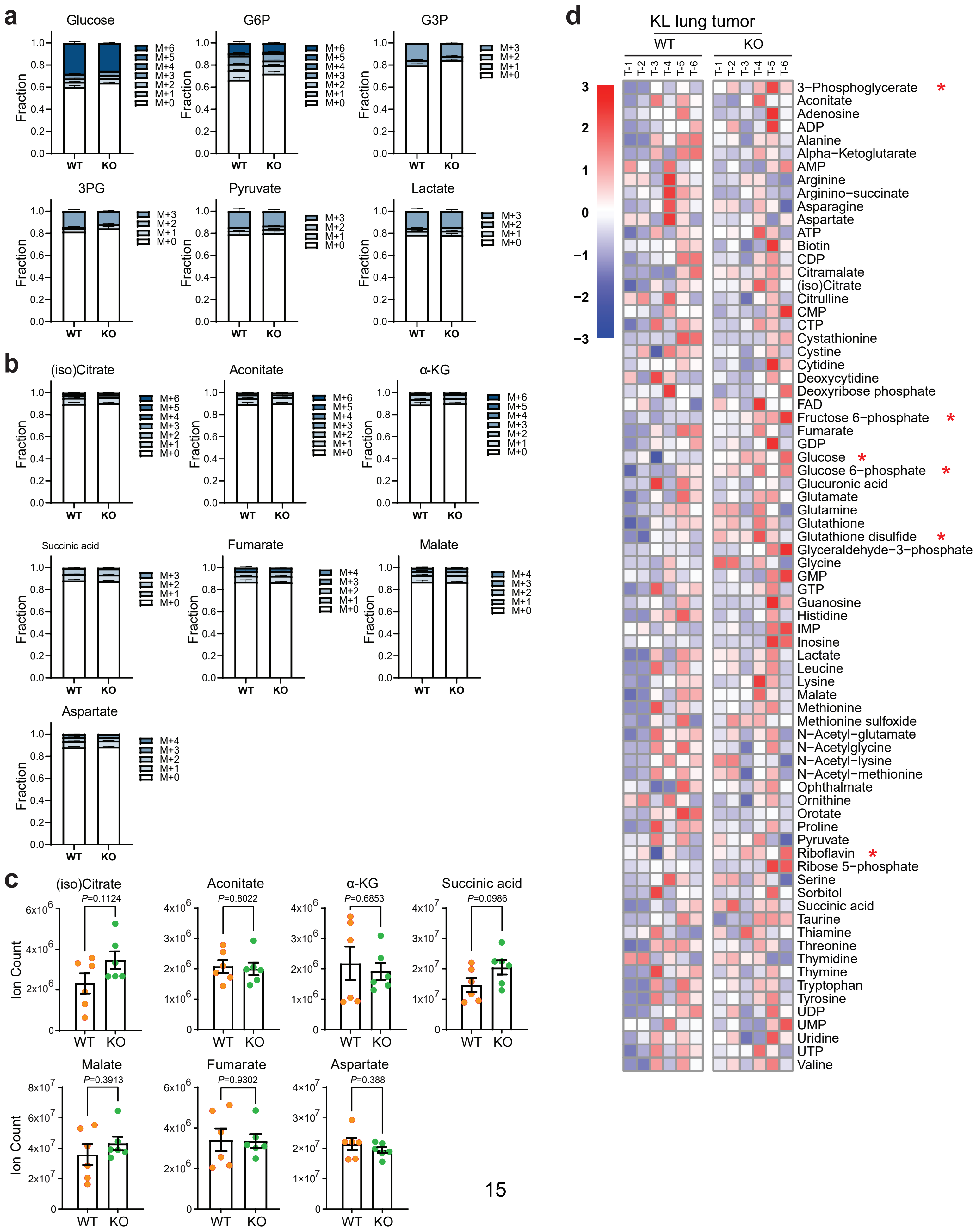

Supplementary Fig. 8: G6PD loss does not affect KL lung tumor TCA cycle metabolism.

a. ^{13}C labeling fraction of glycolytic intermediates from glucose of *G6pd^{WT};KL* (n = 6 mice) and *G6pd^{KO};KL* (n = 6 mice) lung tumors in fasted state (food was removed from the mice at approximately 9:00AM, and mice were euthanized with samples collected at 3:00PM) at 12 weeks post-tumor induction. Glucose 6-phosphate (G6P), glyceraldehyde 3-phosphate (G3P), 3-phosphoglycerate (3-PG),

b. ^{13}C labeling fraction of TCA cycle intermediates from glucose of tumors same with (a). α-ketoglutarate (α-KG).

c. Pool size of TCA cycle intermediates of tumors same with (a).

d. A heatmap of water-soluble metabolites of tumors same with (a).

Data are presented as mean ± SEM, significance was calculated by two-tailed unpaired *t*-test (c). *$P < 0.05$. Source data are provided as a Source Data file.

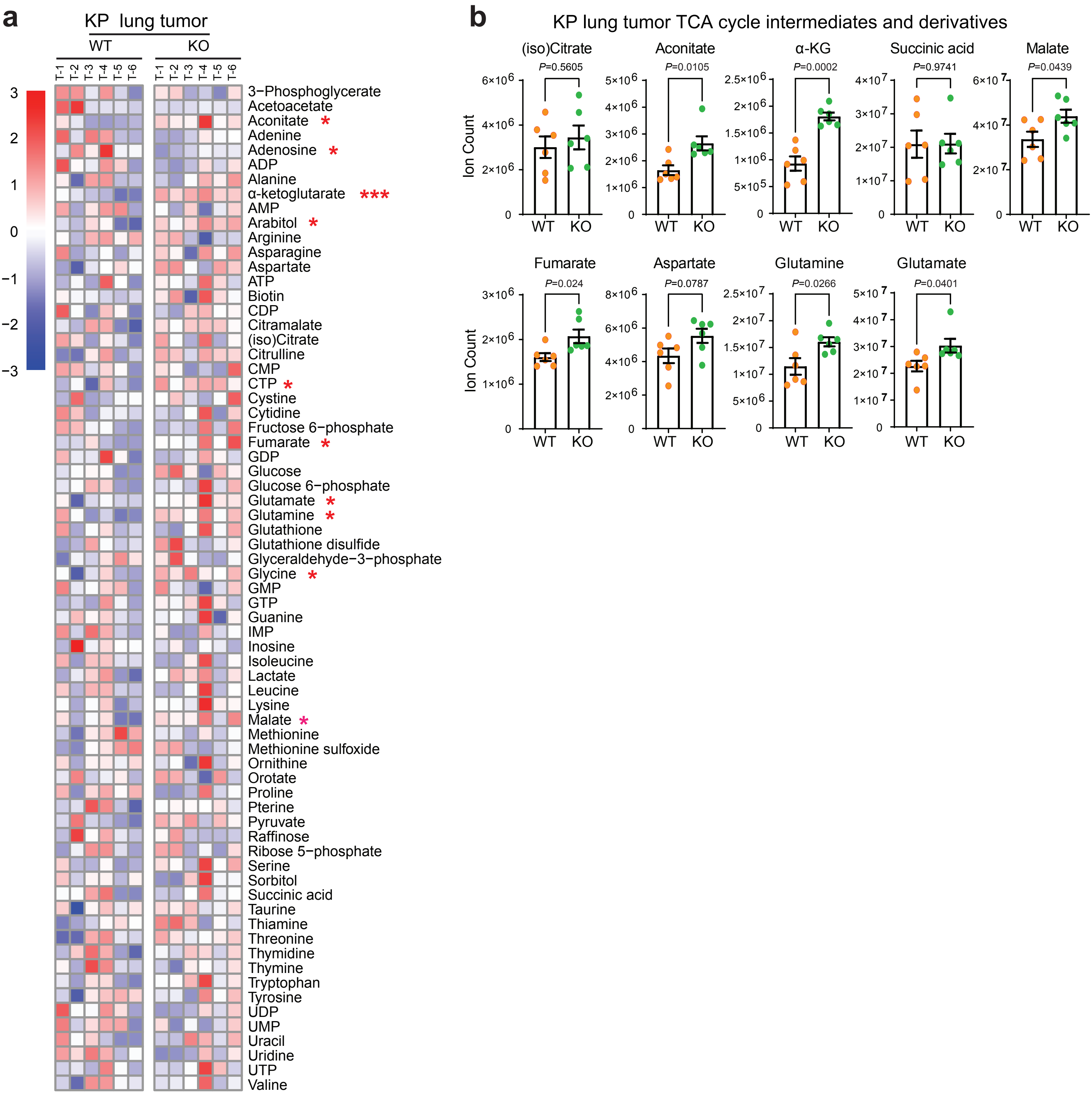
a
KP lung tumor
WT
KO
T-1
T-2
T-3
T-4
T-5
T-6
3
2
1
0
−1
−2
−3
3−Phosphoglycerate
Acetoacetate
Aconitate *
Adenine
Adenosine *
ADP
Alanine
α-ketoglutarate ***
AMP
Arabitol *
Arginine
Asparagine
Aspartate
ATP
Biotin
CDP
Citramalate
(iso)Citrate
Citrulline
CMP
CTP *
Cystine
Cytidine
Fructose 6−phosphate
Fumarate *
GDP
Glucose
Glucose 6−phosphate
Glutamate *
Glutamine *
Glutathione
Glutathione disulfide
Glyceraldehyde−3−phosphate
Glycine *
GMP
GTP
Guanine
IMP
Inosine
Isoleucine
Lactate
Leucine
Lysine
Malate *
Methionine
Methionine sulfoxide
Ornithine
Orotate
Proline
Pterine
Pyruvate
Raffinose
Ribose 5−phosphate
Serine
Sorbitol
Succinic acid
Taurine
Thiamine
Threonine
Thymidine
Thymine
Tryptophan
Tyrosine
UDP
UMP
Uracil
Uridine
UTP
Valine
b
KP lung tumor TCA cycle intermediates and derivatives
(iso)Citrate
P=0.5605
Aconitate
P=0.0105
α-KG
P=0.0002
Succinic acid
P=0.9741
Malate
P=0.0439
Fumarate
P=0.024
Aspartate
P=0.0787
Glutamine
P=0.0266
Glutamate
P=0.0401
Ion Count
WT
KO

Supplementary Fig. 9: G6PD loss increases the pool size levels of TCA cycle intermediates in KP lung tumors.

a. Heatmap of water-soluble metabolites of *G6pd*^{WT}*;KP* (n = 6 mice) and *G6pd*^{KO}*;KP* (n = 6 mice) lung tumors in fasted state (food was removed from the mice at approximately 9:00AM, and mice were euthanized with samples collected at 3:00PM) at 12 weeks post-tumor induction.

b. Pool size of TCA cycle intermediates and derivatives of *G6pd*^{WT}*;KP* and *G6pd*^{KO}*;KP* tumors from (a). α-ketoglutarate (α-KG).

Data are presented as mean ± SEM, significance was calculated by two-tailed unpaired *t*-test (a, b). **P*< 0.05; ****P*< 0.001. Source data are provided as a Source Data file.

Supplementary Fig. 10

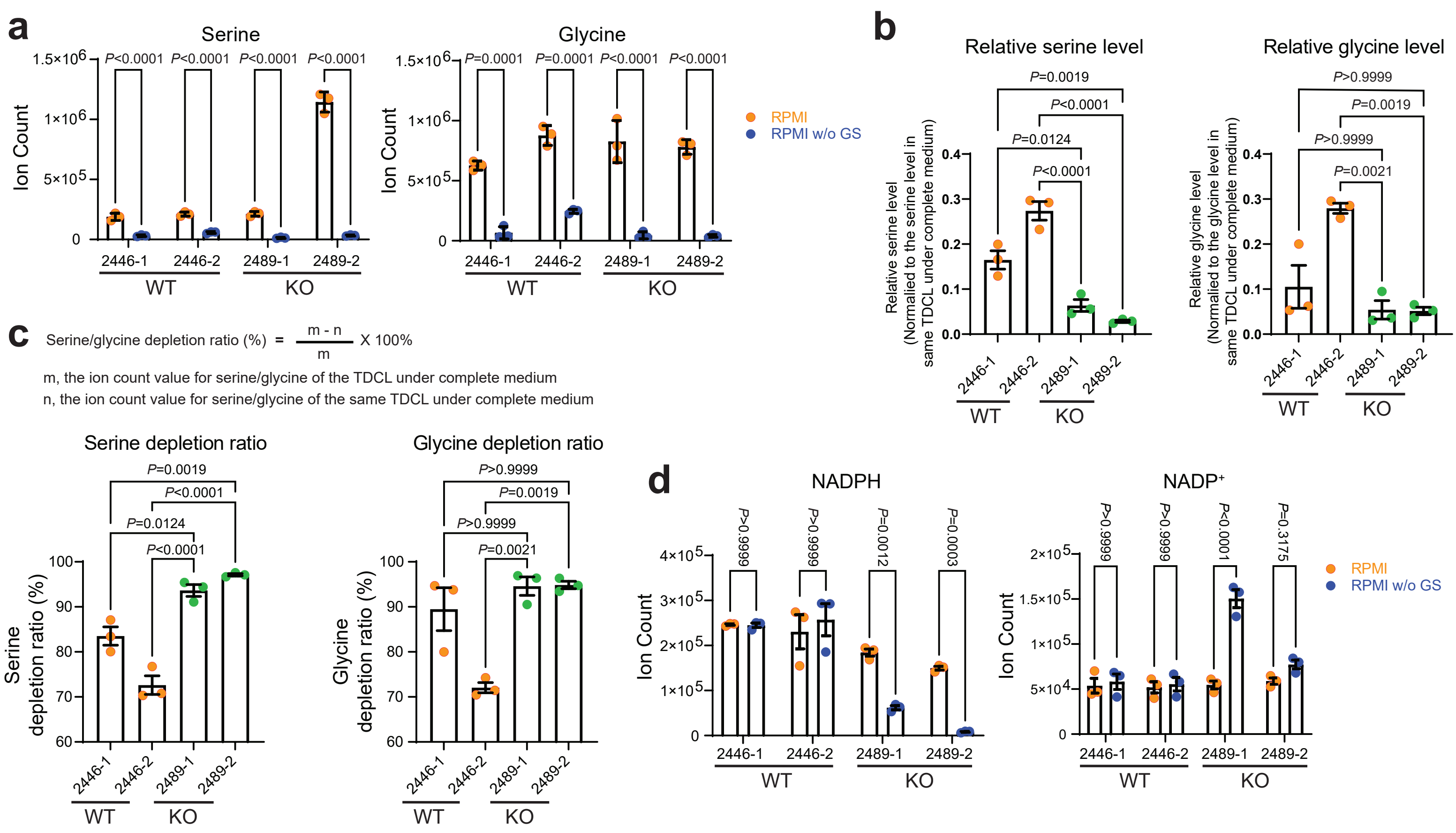

Supplementary Fig. 10: Serine/glycine depletion in KL TDCLs.

a. Pool size of intracellular serine and glycine of *G6pd*^{WT}*;KL* and *G6pd*^{KO}*;KL* TDCLs after 24 hours cultured in RPMI medium with or without serine and glycine. RPMI denotes cells cultured in complete RPMI medium, RPMI w/o GS denotes cells cultured in complete RPMI medium without serine and glycine. n = 3 replicates for each clone under different conditions.

b. Relative intracellular serine and glycine pool size levels of *G6pd*^{WT}*;KL* and *G6pd*^{KO}*;KL* TDCLs after 24 hours cultured in RPMI medium without serine and glycine. RPMI denotes cells cultured in complete RPMI medium, RPMI w/o GS denotes cells cultured in complete RPMI medium without serine and glycine. n = 3 replicates for each clone under different conditions.

c. Intracellular serine and glycine depletion ratio of *G6pd*^{WT}*;KL* and *G6pd*^{KO}*;KL* TDCLs after 24 hours cultured in RPMI medium without serine/glycine depleted medium. RPMI denotes cells cultured in complete RPMI medium, RPMI w/o GS denotes cells cultured in complete RPMI medium without serine and glycine. n = 3 replicates for each clone under different conditions.

d. Pool size levels of NADPH and NADP+ of *G6pd*^{WT}*;KL* and *G6pd*^{KO}*;KL* TDCLs after 24 hours cultured in RPMI medium with or without serine and glycine. RPMI denotes cells cultured in complete RPMI medium, RPMI w/o GS denotes cells cultured in complete RPMI medium without serine and glycine. n = 3 replicates for each clone under different conditions.

Data are presented as mean ± SEM, significance was calculated by two-way ANOVA followed by Bonferroni's multiple comparisons test (a, b, c, d, e). Source data are provided as a Source Data file.

Supplementary Table 1: List of the 28 studies involved in the overall survival analysis for lung cancer patients in the cBioPortal datasets

NO.	Study
1	Lung Adenocarcinoma (Broad, Cell 2012)
2	Lung Adenocarcinoma (CPTAC, Cell 2020)
3	Lung Adenocarcinoma (MSK Mind,Nature Cancer 2022)
4	Lung Adenocarcinoma (MSK, 2021)
5	Lung Adenocarcinoma (MSK, J Thorac Oncol 2020)
6	Lung Adenocarcinoma (MSK, NPJ Precision Oncology 2021)
7	Lung Adenocarcinoma (MSK, Science 2015)
8	Lung Adenocarcinoma (OncoSG, Nat Genet 2020)
9	Lung Adenocarcinoma (TCGA, Firehose Legacy)
10	Lung Adenocarcinoma (TCGA, Nature 2014)
11	Lung Adenocarcinoma (TCGA, PanCancer Atlas)
12	Lung Adenocarcinoma (TSP, Nature 2008)
13	Lung Adenocarcinoma Met Organotropism (MSK, Cancer cell 2023)
14	Lung Cancer (SMC, Cancer Research 2016)
15	Lung Cancer in Never Smokers (NCI, Nature Genetics 2021)
16	Lung Squamous Cell Carcinoma (CPTAC, Cell 2021)
17	Lung Squamous Cell Carcinoma (TCGA, Firehose Legacy)
18	Lung Squamous Cell Carcinoma (TCGA, Nature 2012)
19	Lung Squamous Cell Carcinoma (TCGA, PanCancer Atlas)
20	Metastatic Non-Small Cell Lung Cancer (MSK, Nature Medicine 2022)
21	Non-Small Cell Cancer (MSK, Cancer Discov 2017)
22	Non-Small Cell Lung Cancer (MSK, Cancer Cell 2018)
23	Non-Small Cell Lung Cancer (MSK, J Clin Oncol 2018)
24	Non-Small Cell Lung Cancer (MSK, Science 2015)
25	Non-Small Cell Lung Cancer (TRACERx, NEJM & Nature 2017)
26	Non-Small Cell Lung Cancer (University of Turin, Lung Cancer 2017)
27	Non-Small Cell Lung Cancer Brain Metastasis (MSK, Nat Commun 2023)
28	Pan-Lung Cancer (TCGA, Nat Genet 2016)

Supplementary Table 2: Sequences of mouse genotyping primers used in this study

*Kras*_F	CCATGGCTTGAGTAAGTCTGC
*Kras*_R	CGCAGACTGTAGAGCAGCG
*G6pd*_F	TCCTGAGTACTGGAATCACAACTG
*G6pd*_R	CACAGAACAGATGCTCATTGTGAG
*Lkb1*_F	ATCGGAATGTGATCCAGCTT
*Lkb1*_R	ACGTAGGCTGTGCAACCTCT
*P53*_F	GGTTAAACCCAGCTTGACCA
*P53*_R	GGAGGCAGAGACAGTTGGAG
