# Supplementary material for: Glucose-6-phosphate dehydrogenase maintains redox homeostasis and biosynthesis in LKB1-deficient KRAS-driven lung cancer
Source: Nat Commun. 2024 Jul 12;15:5857. doi: 10.1038/s41467-024-50157-8 (PMC11245543; doi:10.1038/s41467-024-50157-8)
Supplement: Supplementary file 3 — Description of Additional Supplementary Information [file 41467_2024_50157_MOESM3_ESM.docx]

**Descriptions of Additional Supplementary File**

**File Name:** Supplementary Data 1

**Description:** RNA-Seq data for *G6pd^KO^;KL* and *G6pd^WT^;KL* lung tumor samples.
