# Supplementary material for: Glucose-6-phosphate dehydrogenase maintains redox homeostasis and biosynthesis in LKB1-deficient KRAS-driven lung cancer
Source: Nat Commun. 2024 Jul 12;15:5857. doi: 10.1038/s41467-024-50157-8 (PMC11245543; doi:10.1038/s41467-024-50157-8)
Supplement: Supplementary file 5 — Reporting Summary [file 41467_2024_50157_MOESM5_ESM.pdf]

nature portfolio

Corresponding author(s): Jessie Yanxiang Guo

Last updated by author(s): Jun 3, 2024

Reporting Summary

Nature Portfolio wishes to improve the reproducibility of the work that we publish. This form provides structure for consistency and transparency in reporting. For further information on Nature Portfolio policies, see our Editorial Policies and the Editorial Policy Checklist.

Statistics

For all statistical analyses, confirm that the following items are present in the figure legend, table legend, main text, or Methods section.

n/a	Confirmed	
☐	☒	The exact sample size (*n*) for each experimental group/condition, given as a discrete number and unit of measurement
☐	☒	A statement on whether measurements were taken from distinct samples or whether the same sample was measured repeatedly
☐	☒	The statistical test(s) used AND whether they are one- or two-sided<br>*Only common tests should be described solely by name; describe more complex techniques in the Methods section.*
☒	☐	A description of all covariates tested
☐	☒	A description of any assumptions or corrections, such as tests of normality and adjustment for multiple comparisons
☐	☒	A full description of the statistical parameters including central tendency (e.g. means) or other basic estimates (e.g. regression coefficient) AND variation (e.g. standard deviation) or associated estimates of uncertainty (e.g. confidence intervals)
☐	☒	For null hypothesis testing, the test statistic (e.g. *F*, *t*, *r*) with confidence intervals, effect sizes, degrees of freedom and *P* value noted<br>*Give P values as exact values whenever suitable.*
☒	☐	For Bayesian analysis, information on the choice of priors and Markov chain Monte Carlo settings
☒	☐	For hierarchical and complex designs, identification of the appropriate level for tests and full reporting of outcomes
☒	☐	Estimates of effect sizes (e.g. Cohen's *d*, Pearson's *r*), indicating how they were calculated

Our web collection on statistics for biologists contains articles on many of the points above.

Software and code

Policy information about availability of computer code

Data collection	cBioPortal, Visiopharm A/S.
Data analysis	cBioPortal, GraphPad Prism 9.1.0, GSEA_4.3.2, ImageJ 1.52a, Visiopharm A/S, AccuCor written in R (doi: 10.1021/acs.analchem.7b00396).

For manuscripts utilizing custom algorithms or software that are central to the research but not yet described in published literature, software must be made available to editors and reviewers. We strongly encourage code deposition in a community repository (e.g. GitHub). See the Nature Portfolio guidelines for submitting code & software for further information.

Data

Policy information about availability of data

All manuscripts must include a data availability statement. This statement should provide the following information, where applicable:

- Accession codes, unique identifiers, or web links for publicly available datasets
- A description of any restrictions on data availability
- For clinical datasets or third party data, please ensure that the statement adheres to our policy

The raw data of mRNA-seq have been deposited in the Gene Expression Omnibus (GEO) database under accession number GSE253613 [https://www.ncbi.nlm.nih.gov/geo/query/acc.cgi?acc=GSE253613]. Additionally, full list of RNA-seq result is provided in Supplementary Data 1. The metabolomics and lipidomics raw data have been deposited in Metabolomics Workbench under project ID PR001843 [https://www.metabolomicsworkbench.org/data/DRCCMetadata.php?Mode=Project&ProjectID=PR001843]. Source data are provided with this paper.

Research involving human participants, their data, or biological material

Policy information about studies with human participants or human data. See also policy information about sex, gender (identity/presentation), and sexual orientation and race, ethnicity and racism.

Reporting on sex and gender	N/A
Reporting on race, ethnicity, or other socially relevant groupings	N/A
Population characteristics	N/A
Recruitment	N/A
Ethics oversight	N/A

Note that full information on the approval of the study protocol must also be provided in the manuscript.

Field-specific reporting

Please select the one below that is the best fit for your research. If you are not sure, read the appropriate sections before making your selection.

[x] Life sciences [ ] Behavioural & social sciences [ ] Ecological, evolutionary & environmental sciences

For a reference copy of the document with all sections, see nature.com/documents/nr-reporting-summary-flat.pdf

Life sciences study design

All studies must disclose on these points even when the disclosure is negative.

Sample size	Sample sizes were chosen based on the power calculation.
Data exclusions	No data exclusions.
Replication	Experimental findings were replicated with at least 3 independent biological repeats.
Randomization	Mice were randomly assigned to different treatment groups.
Blinding	The investigators were blinded to the group allocation during experiments and when assessing outcomes.

Reporting for specific materials, systems and methods

We require information from authors about some types of materials, experimental systems and methods used in many studies. Here, indicate whether each material, system or method listed is relevant to your study. If you are not sure if a list item applies to your research, read the appropriate section before selecting a response.

Materials & experimental systems

n/a	Involved in the study	
[ ]	[x]	Antibodies
[ ]	[x]	Eukaryotic cell lines
[x]	[ ]	Palaeontology and archaeology
[ ]	[x]	Animals and other organisms
[x]	[ ]	Clinical data
[x]	[ ]	Dual use research of concern
[x]	[ ]	Plants

Methods

n/a	Involved in the study	
[x]	[ ]	ChIP-seq
[x]	[ ]	Flow cytometry
[x]	[ ]	MRI-based neuroimaging

Antibodies

Antibodies used	anti-G6PD (Abcam, Cata#AB993, Clone#Polyclonal, Lot#GR274589-46, 1:2000 dilution), anti-Ki67 (Abcam, Cata#ab15580, Clone#Polyclonal, Lot# GR3375556-1, 1:2000 dilution), anti-pS6 (Cell Signaling, Cata#4858S, Clone#D57.2.2E, Lot#21, 1:500 dilution), anti-P-p42/44 MAPK (pERK) (Cell Signaling, Cata#9101S, Clone#NA, Lot# 26, 1:500 dilution), anti-cleaved caspase3 (Cell Signaling, Cata#9661S, Clone#NA, Lot#47, 1:150 dilution), anti-p53 (Leica, Cata#NCL-L-p53-CM5p, Clone# POLYCLONAL, Lot#6065476, 1:2000 dilution), anti-p21 (Santa Cruz Biotech, Cata#sc-6246, Clone# F-5, Lot#I1020, 1:1000 dilution), anti-8-oxo-dG (R&D systems, Cata#4354-MC-050, Clone#15A3, Lot#P323432, 1:500 dilution), anti-γ-H2AX (Cell Signaling, Cata#9718, Clone#20E3, Lot#21, 1:1000 dilution), anti-NQO1 (Invitrogen, Cata#PA5-21290, Clone#AB_11153144, Lot#YL4152869, 1:1000 dilution), anti-NRF2 (Invitrogen,

Cata#PA5-27882, Clone#AB_2545358, Lot#YF3956921A, 1:1000 dilution), anti-pACC (S79) (Cell Signaling, Cata#3661, Clone#NA, Lot#10, 1:1000 dilution), anti-pAMPK (Cell Signaling, Cata#50081, Clone#D4D6D, Lot#6, 1:1000 dilution).

Validation

Commercial per-validated antibodies were purchased from reputable sources , with validation data present on the manufacturer websites as listed blow: G6PD (https://www.abcam.com/products/primary-antibodies/glucose-6-phosphate-dehydrogenase-antibody-ab993.html), Ki67 (https://www.abcam.com/products/primary-antibodies/ki67-antibody-ab15580.html), pS6 (https://www.cellsignal.com/products/primary-antibodies/phospho-s6-ribosomal-protein-ser235-236-d57-2-2e-xp-rabbit-mab/4858), P-p42/44 MAPK (pERK) (https://www.cellsignal.com/products/primary-antibodies/phospho-p44-42-mapk-erk1-2-thr202-tyr204-antibody/9101), cleaved caspase3 (https://www.cellsignal.com/products/primary-antibodies/cleaved-caspase-3-asp175-antibody/9661), p53 (https://shop.leicabiosystems.com/us/ihc-ish/ihc-primary-antibodies/pid-p53-protein-cm5), p21 (https://www.scbt.com/p/p21-antibody-f-5), 8-oxo-dG (https://www.rndsystems.com/products/8-oxo-dg-antibody-15a3_4354-mc-050), γ-H2AX (https://www.cellsignal.com/products/primary-antibodies/phospho-histone-h2a-x-ser139-20e3-rabbit-mab/9718), NQO1 (https://www.thermofisher.com/antibody/product/NQO1-Antibody-Polyclonal/PA5-21290), NRF2 (https://www.thermofisher.com/antibody/product/Nrf2-Antibody-Polyclonal/PA5-27882), pACC (S79) (https://www.cellsignal.com/products/primary-antibodies/phospho-acetyl-coa-carboxylase-ser79-antibody/3661), pAMPK (https://www.cellsignal.com/products/primary-antibodies/phospho-ampka-thr172-d4d6d-rabbit-mab/50081).

Eukaryotic cell lines

Policy information about cell lines and Sex and Gender in Research

Cell line source(s)	All cell lines in this study were generated by ourselves. Cell lines 2446-1 and 2446-2 were derived from male mice with G6pdWT-KL lung tumor. Cell lines 2489-1 and 2489-2 were derived from male mice with G6pdKO-KL lung tumor.
Authentication	All cell lines were confirmed to have KrasG12D mutation and Lkb1 deficiency. Cell lines 2446-1 and 2446-2 were confirmed for G6pd expression, while cell lines 2489-1 and 2489-2 were confirmed for G6pd deficiency.
Mycoplasma contamination	All cell lines used in this study were tested negative for mycoplasma contamination.
Commonly misidentified lines (See ICLAC register)	No commonly misidentified cell lines were in this study.

Animals and other research organisms

Policy information about studies involving animals; ARRIVE guidelines recommended for reporting animal research, and Sex and Gender in Research

Laboratory animals	Wild type male C57BL6/J mice (Stock No: 008463) were obtained from the Jackson Laboratory. G6pd+/+;KrasLSL-G12D;Lkb1flox/flox mice, G6pd+/+;KrasLSL-G12D;P53flox/flox mice were generated in our lab's previous study. G6pdflox/flox mice were obtained from Rutgers Cancer Institute of New Jersey Genome Editing core facility. G6pdflox/flox;KrasLSL-G12D;Lkb1flox/flox mice were generated by cross-breeding G6pdflox/flox mice with G6pd+/+;KrasLSL-G12D;Lkb1flox/flox mice, G6pdflox/flox;KrasLSL-G12D;P53flox/flox mice were generated by cross-breeding G6pdflox/flox mice with G6pd+/+;KrasLSL-G12D;P53flox/flox mice, G6pd+/+;KrasLSL-G12D;P53flox/flox;Lkb1flox/flox mice were generated by cross-breeding G6pd+/+;KrasLSL-G12D;Lkb1flox/flox mice with G6pd+/+;KrasLSL-G12D;P53flox/flox mice, G6pdflox/flox;KrasLSL-G12D;P53flox/flox;Lkb1flox/flox mice were generated by cross-breeding G6pdflox/flox mice with G6pd+/+;KrasLSL-G12D;P53flox/flox;Lkb1flox/flox. Both male and female mice at 6-8 weeks old were used in this study.
Wild animals	No wild animals were used in this study.
Reporting on sex	We compared the response to G6PD loss between male and female mice bearing KL lung tumors. The results showed no significant difference, which has not been included in the data collection. Both male and female mice were used in this study.
Field-collected samples	This study didn't involve samples collected from the field.
Ethics oversight	All animal experiments were approved by the Institutional Animal Care and Use Committee of Rutgers University

Note that full information on the approval of the study protocol must also be provided in the manuscript.

Plants

Seed stocks	N/A
Novel plant genotypes	N/A
Authentication	N/A
